# Simplified LC-MS Method for Analysis of Sterols in Biological Samples

**DOI:** 10.3390/molecules25184116

**Published:** 2020-09-09

**Authors:** Cene Skubic, Irena Vovk, Damjana Rozman, Mitja Križman

**Affiliations:** 1 Center for Functional Genomics and Bio-Chips, Institute of Biochemistry, Faculty of Medicine, University of Ljubljana, Zaloška cesta 4, SI-1000 Ljubljana, Slovenia; cene.skubic@mf.uni-lj.si (C.S.); damjana.rozman@mf.uni-lj.si (D.R.); 2 Department of Food Chemistry, National Institute of Chemistry, Ljubljana, Hajdrihova 19, SI-1000 Ljubljana, Slovenia; irena.vovk@ki.si

**Keywords:** cholesterol, LC-MS, sterol intermediates, cholesterol synthesis, pentafluorophenyl, PFP

## Abstract

We developed a simple and robust liquid chromatographic/mass spectrometric method (LC-MS) for the quantitative analysis of 10 sterols from the late part of cholesterol synthesis (zymosterol, dehydrolathosterol, 7-dehydrodesmosterol, desmosterol, zymostenol, lathosterol, FFMAS, TMAS, lanosterol, and dihydrolanosterol) from cultured human hepatocytes in a single chromatographic run using a pentafluorophenyl (PFP) stationary phase. The method also avails on a minimized sample preparation procedure in order to obtain a relatively high sample throughput. The method was validated on 10 sterol standards that were detected in a single chromatographic LC-MS run without derivatization. Our developed method can be used in research or clinical applications for disease-related detection of accumulated cholesterol intermediates. Disorders in the late part of cholesterol synthesis lead to severe malformation in human patients. The developed method enables a simple, sensitive, and fast quantification of sterols, without the need of extended knowledge of the LC-MS technique, and represents a new analytical tool in the rising field of cholesterolomics.

## 1. Introduction

Cholesterol is the most abundant sterol molecule in animal cells. It is an important part of the cell membrane, where also the majority of cholesterol is located. In cell membranes, cholesterol is responsible for proper physical properties like fluidity and rigidness and is a part of lipid rafts [1]. Besides its role in membranes, cholesterol is the precursor for bile acids and oxysterols, and its synthesis metabolites are vitamin D precursors. Cholesterol is synthesized in the first step from acetyl-CoA through a mevalonate pathway to squalene. The mevalonate pathway of synthesis is well known and statins, widely used drugs for treating hypercholesterolemia, affect the rate-limiting enzyme HMG-CoA reductase which transforms HMG-CoA to mevalonate. Lanosterol is the first molecule in the post-squalene part of cholesterol synthesis and the first molecule with a characteristic sterol ring [2,3]. From lanosterol on, the synthesis is separated into the proposed Bloch and Kandutsch–Russell pathways, schematically represented in Ačimovič et al. [3]. The final step in the Bloch branch is the conversion of desmosterol to cholesterol by sterol-Δ24-reductase (DHCR24). However, because all sterols intermediated downstream from lanosterol have a double bond on position C24, DHCR24 can act already on lanosterol which is proposed in the Kandutsch–Russell branch. In this case, all intermediates from 24,25-dihydrolanosterol to 7-dehydrocholesterol contain a saturated side chain and cholesterol is synthesized in the last step from 7-dehydrocholesterol by the DHCR7 enzyme. DHCR24 can theoretically transform any cholesterol synthesis intermediate from lanosterol on; the two branches cannot be treated separately. In both pathways combined, there are at least 20 different sterol molecules, from which many of them share the same molecule mass and have a very similar structure [4]. However, the theoretical number of sterol intermediates from lanosterol to cholesterol is much higher [5] but not all of these sterols have been detected in biological systems.

Cholesterol synthesis is a housekeeping pathway and any abnormalities in the late part of synthesis usually lead to accumulation of sterol intermediates which results in severe malformations in humans [6]. Examples of diseases include desmosterolosis (desmosterol accumulation), lathosterolosis (lathosterol accumulation), Conradi–Huenermann–Happle syndrome (zymosterol accumulation), Smith–Lemli–Opitz syndrome, or Antley–Bixler syndrome (lanosterol and 24,25-dihydrolanosterol accumulation) [6,7]. In recent years, studies have shown that sterol intermediates, apart from being cholesterol precursors, have other important physiological functions, like a role in spermatogenesis, oligodendrocyte remyelination, and activations of nuclear receptors [8,9,10]. Due to the utmost importance for further cholesterol metabolism research, a new research field has arisen, named “cholesterolomics” [11]. Cholesterolomics can be regarded as the identification and quantification of cholesterol, its post squalene precursors, and metabolites of cholesterol and of its precursors, in biological samples. To decipher the physiological function of sterol intermediates and detect accumulating sterols in different pathologies for a given situation or experimental setup, the need for a customized analytical methodology might therefore arise (as in the given case), preferably providing a simple and straightforward procedure.

Chromatographic analysis of sterols can be accomplished in a variety of ways. Since many sterols share the same molecular mass and have a very similar structure, discriminating mass spectrometric detection is challenging or even impossible, especially with compounds which are prone to abundant fragmentation [12]. Likewise, a good chromatographic resolution can also be problematic due to their very similar chromatographic behavior. Similarly, a situation, albeit a bit less challenging, can also be encountered when oxysterols, slightly more polar analytes, are analyzed on their own. As a result, analytics of oxysterols alone have been a subject of substantial research as well [13]. Traditionally, gas chromatography (GC) has been used as the technique of choice when an improved resolution in sterol analysis was needed. Sterol detection in GC analysis can be done by either flame-ionization (FID) or mass spectrometric (MS) detection. Sterols can be analyzed by GC in both underivatized [14] or derivatized form [15,16]. In the latter case, there is some improvement in chromatographic resolution. However, the derivatization step involves a few drawbacks. It is not only much more labor-intensive but also introduces other sources of error into the analytical procedure. Nonetheless, the GC-MS technique is also inherently less sensitive compared to LC-MS. Therefore, the analysis of sterols by high-performance liquid chromatography (HPLC) is a valid and practical alternative. LC-MS has become the technique of choice also in the field of sterol analytics, steadily displacing established techniques like gas chromatography [17].

There are a number of published HPLC methods for sterol analysis. Since sterols have a low inherent UV detection response, UV detection is used only in cases when analyte concentrations are relatively high. In cases when minor sterol compounds, e.g., sterol intermediates, need to be analyzed, MS detection is likely to be the only viable option. Since sterols are not ionizable compounds, electrospray ionization (ESI) should not be a sensible option in MS detection. However, under some very narrow conditions, sterols can be ionized in the ESI source in the form of adducts, as evidenced in a few publications [18,19,20]. Unfortunately, under most other chromatographic conditions, ESI ionization is not a good option. In most other cases, as in the presented work, atmospheric-pressure chemical ionization (APCI) is the most common ionization technique for sterols [18,21,22,23]. They can also be successfully ionized under atmospheric-pressure photo-ionization (APPI) conditions when such an ion source is available [24]. For a variety of reasons, mainly practical ones, most of these methods use reversed-phase columns, and predominantly the octadecyl-silica type of stationary phase [17,23,25,26]. Many of them involve gradient elution in order to improve selectivity. Low column temperatures are also commonly employed to accentuate the minute differences among individual sterols and therefore enhance the overall chromatographic resolution.

The aim of our study was to develop a simple and robust LC-MS method that would allow the separation and quantification of 10 structurally similar sterols from cholesterol synthesis in a single chromatographic run. The LC-MS method in the presented work provides sterol separation using a less common column phase, namely pentafluorophenyl (PFP) stationary phase, and avails on a different set of retention mechanisms usually found in common reversed phases [27].

## 2. Results and Discussion

### 2.1. Chromatographic Separation and Detection

As a starting point, we deliberately avoided procedures involving sample derivatization [28], due to the drawbacks mentioned previously. Therefore, we opted to use a well-known and proven methodology, like the one published by McDonald et al. (2007) [26], since it is capable of resolving most of the analytes of interest in the underivatized form, and using similar instrumentation. However, the largest pitfall is a relatively poor inherent sensitivity of this method for some of the compounds of interest, e.g., zymosterol, desmosterol, and lathosterol [26]. Additionally, we have not obtained satisfactory separation for some other compounds of interest, not covered by this paper. Even later developments from the same group [23] gave no satisfactory separation between lathosterol and cholesterol, the former being an important parameter in the analysis of biological samples. Therefore, we opted to develop a custom methodology for sterols alone, without involving the analytics of oxysterols. The goal was also to improve sensitivity as much as possible, in order to avoid the need for further sample purification and/or concentration.

**Figure 1:**
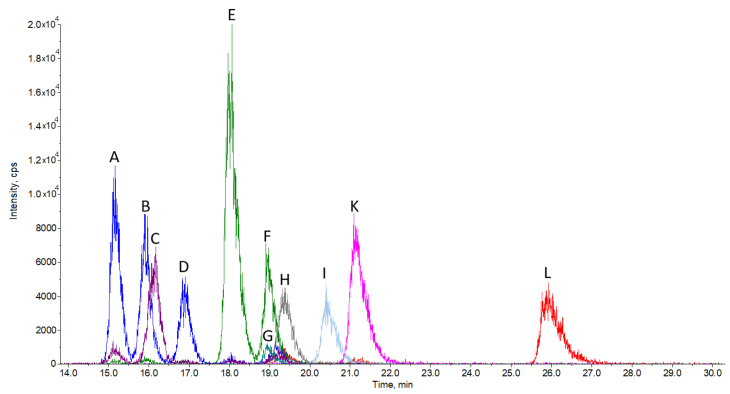
Chromatogram of sterol standards: zymosterol (A), 24-dehydrolathosterol (B), 7-dehydrodesmosterol (C), desmosterol (D), zymostenol (E), lathosterol (F), lathosterol-d7 (G), FFMAS (H), TMAS (I), lanosterol (K), and dihydrolanosterol (L). The peak (J) is dedicated to cholesterol and is not presented in this chromatogram. Cholesterol is not assayed, since its concentration is several orders of magnitude larger than other sterols. Cholesterol was assayed using a separate analytical method. Separation conditions are described in Section 3.3 LC-MS Analysis.

**Figure 2:**
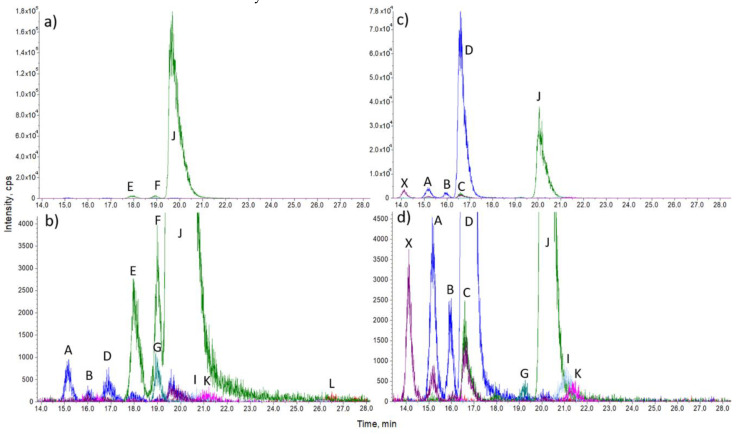
Representative chromatograms of sterols isolated from native HepG2 cells (**a**,**b**) and HepG2 DHCR KO cells (**c**,**d**). Each chromatogram is presented in two magnifications to account for a higher quantity of cholesterol compared to intermediate sterols (**a**,**b**) or desmosterol and cholesterol compared to other sterols (**c**,**d**). Zymosterol (A), 24-dehydrolathosterol (B), 7-dehydrodesmosterol (C), 7-desmosterol (D), zymostenol (E), F-lathosterol (F), lathosterol-d7 (G), TMAS (I), holesterol (J), lanosterol (K), dihydrolanosterol (L), and an unknown peak with the MRM of 365/199 (X). All peaks with the same MRM transition (A, B, D and E, F, J) are chromatographically separated. Peak of cholesterol is depicted for illustrative purpose only.

We were able to chromatographically separate 10 sterols using a pentafluorophenyl stationary phase column and find specific multiple reaction monitoring mode (MRM) transitions for their quantification (Figure 1). To ensure successful chromatographic separation, the analysis run time is rather long, namely 30 min, in order to obtain sufficient resolution, as is in the case of most similar published methods. The relatively long run time is also partially due to the isocratic elution used, but in turn, there are no issues with long column re-equilibration times, as it would be in the case of gradient elution. Being isocratic, the method can also be scaled up for preparative separation purposes without gradient elution-associated difficulties. All sterols elute in the time interval between 15 and 27 min. The most challenging to separate were zymosterol, 24-dehydrolathosterol, and desmosterol, which all have a molecular weight of 384.6 and the same MRM transition (Table 3) of 367/215. A good chromatographic separation is therefore a must in order to separately detect and quantify each of these sterols. A similar problem had to be solved for the successful separation of zymostenol and lathosterol (Figure 1) with MRM 369/215 and cholesterol in the case of sample analysis (Figure 2).

The mobile phase consisting of water, methanol, and 1-propanol, in combination with the pentafluorophenyl stationary phase (PFP), proved to be a good combination since selectivity and the overall resolution was even slightly improved at higher column temperatures, which is usually not the case in most HPLC methodologies used in sterol analysis. The validation of the method was done on different concentrations of standards and in different times of injection (as described in Materials and Methods). Validation data are represented in Table 1 and described in Section 3.4.

### 2.2. Sample Analysis

**Figure 3:**
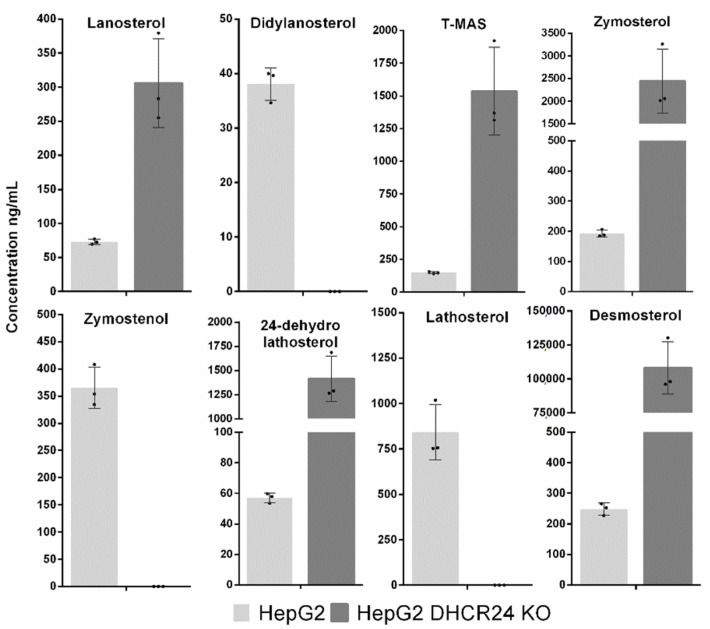
Concentrations of sterol intermediates isolated from HepG2 and HepG2 DHCR-KO cell lines. All concentrations are calculated as ng/mL per 10^7^, to normalize sterol concentration based on the number of counted cells. All data are represented as mean with SD, black dots represent individual measurements.

To test the LC-MS method on biological samples, we isolated sterols from cell cultures as described in the sample preparation. We used hepatocellular cancer cell lines HepG2 and HepG2 with CRISPR-Cas9-generated deletion in the DHCR24 enzyme. In wild type HepG2 cells, we were able to detect (Figure 2) and quantify (Figure 3 and Table 2) eight sterol intermediates. The concentration of sterol FF-MAS in these cells was too low for detection, which is consistent with previously observed FF-MAS concentrations in liver tissue [29,30]. In HepG2 DHCR-KO, all measured sterols are statistically different compared to non-modified HepG2 cells (Table 3). Due to an inactive DHCR24 enzyme, sterol intermediates from the Bloch branch of cholesterol synthesis accumulate in very high concentrations and sterols from the Kandutsch–Russell branch are absent. In the DHCR-KO sample, we also detected an unknown sterol peak (X on Figure 2d) with retention time of 14.15 min and MRM 365/199. This could be one of the sterols from the Bloch pathway for which commercial standards are not available or a sterol intermediate that is normally not present in cells and is the result of further enzymatic transformation.

## 3. Materials and Methods

### 3.1. Materials

All sterol standards (listed in Table 3) were bought from Avanti Polar Lipids (Alabaster, AL, USA), except cholesterol, which is from Merck (Darmstadt, Germany). Chloroform, cyclohexane, 1-propanol, and methanol (all LC-MS grade) were purchased from Honeywell (Charlotte, NC, USA). Dulbecco’s modified Eagle’s medium (DMEM), Penicillin-Streptomycin (P/S), Trypsin, and Fetal Bovine Serum (FBS) were bought from Merck (Darmstadt, Germany).

### 3.2. Sample Preparation

#### 3.2.1. Isolation and Culture of Human Hepatocytes

Here, 1 × 10^6^ of HepG2 and HepG2 with DHCR24 deletion (ordered from Synthego, Menalo Park, CA, USA) cells were plated on a T75 culture flask for each sterol isolation. Cells were plated in classic DMEM medium with 10% FBS and 1% P/S. After 24 h, the cell medium was changed with DMEM with 2% FBS and 1% P/S to lower the cholesterol concentration in the medium and upregulate cholesterol synthesis in the cells. After 48 h, cells were washed two times with Phosphate-buffered saline (PBS) and detached using 1 mL of Trypsin. Detached cells were resuspended in 5 mL of DMEM without FBS and transferred to a 15 mL glass vial with a Polytetrafluoroethylene (PTFE) cap. An amount of 50 µL of cells was used for cell counting on an Adam Automatic Cell Counter (Alpha Metrix Biotech GmbH, Rödermark, Germany). There were between 0.9 and 2.2 × 10^7^ cells counted. The rest of the cells were pelleted by centrifugation at 700 rpm for 5 min and washed one more time with PBS.

#### 3.2.2. Sterol Isolation

Folch reagent (5 mL; 66% chloroform, 33% methanol) was added to each cell pellet and vortexed for 60 s. At this step, 200 ng of internal standard lathosterol-D7 was added and samples were incubated for 2 h at room temperature, with shaking. After incubation, Folch reagent was evaporated using a vacuum centrifuge (Eppendorf Concentrator 5301, Eppendorf, Hamburg, Germany) at 45 °C, followed by hydrolysis with 1 mL of hydrolysis solution (8 g NaOH + 20 mL H_2_O + 180 mL 99.5 % ethanol) at 65 °C with shaking. Extraction was made using 0.5 mL H_2_O and 3 mL of cyclohexane centrifugation on 3500 rpm for 10 min (Eppendorf 5810 R, Eppendorf, Hamburg, Germany). The upper phase was transferred to a new 15 mL vial. Extraction was repeated one more time on the remaining sample with only 3 mL of cyclohexane. The upper phase was pooled and evaporated using a vacuum centrifuge at 45 °C. Samples were resuspended in 300 µL of methanol and transferred to HPLC for analysis.

### 3.3. LC-MS Analysis

Chromatographic separation was done on a Shimadzu Nexera XR HPLC (Shimadzu, Kyoto, Japan) under isocratic conditions, with a mobile phase of methanol/1-propanol/water/formic acid (80:10:10:0.02 %, *v*/*v*/*v*/*v*) and flow of 150 µL/min. Five microliters of standard or sample solutions was injected. Two Phenomenex Luna 3 µm PFP (pentafluorophenyl) columns (Phenomenex, Torrance, CA, USA) connected in series with dimensions 100 × 2 mm and 150 × 2 mm were used for separation, and the oven temperature was set to 40 °C. For detection, a SCIEX Triple Quad™ 3500 mass spectrometer (AB Sciex LLC, Redwood City, CA, USA) was used, with APCI ionization in multiple reaction monitoring mode (MRM). Pairs of parent/daughter ions used in MRM detection have been selected based on daughter ion intensity and the observed signal to noise ratio in order to obtain the maximum sensitivity given the experimental conditions used. Ion source temperature was 350 °C, collision gas was 4, declustering potential was 140 V, curtain gas was 20, entrance potential was 10 V, cell exit potential was 6 V, and collision energy was 45 V. Other detection conditions are displayed in Table 3.

### 3.4. Quantitation, Accuracy, Repeatability, Linearity, and Stability

Injection precision was determined by three injections of a working standard solution containing each sterol standard (except for cholesterol) at 250 ng/mL. Since the cholesterol concentration in samples is significantly higher than other sterols, it was not tested in the method validation as other sterols. Standard solutions with the same sterol concentrations have also been used for the quantification of sterols in samples using the external standard method. Repeatability was assessed by preparing the selected cell culture sample in three replicates using the same sample preparation procedure. Intermediate precision was tested on the same set of samples by analyzing them on three consecutive days. Accuracy has been tested by spiking samples with selected standards and subjecting the spiked samples to the sample preparation procedure, analyzing them, and calculating the recovery. Linearity for all sterols (except for cholesterol) has been tested in the concentration range from 67.5 to 5000 ng/mL. Limits of detection (LOD) and quantitation (LOQ) have been extrapolated from signal to noise measurements obtained for standard solutions used in the linearity tests. Stability was tested in the period of 48 h on selected samples kept refrigerated at 4 °C in the dark.

## 4. Conclusions

In conclusion, we developed a simplified LC-MS method for the detection and quantification of 10 sterols from the late part of cholesterol synthesis using commercially available sterol standards. We were able to chromatographically separate the sterol molecules with the same mass and MRMs. We confirmed our LC-MS method on sterols isolated from HepG2 cell culture samples. Our method can be used also for the quantification of sterols using tissue or serum/plasma samples with minor changes in the isolation protocols described [16,23] or using commercially available kits like MAK175 (Merck, Darmstadt, Germany). Our LC-MS is superior to the previously described GC-MS method [16] and is also able to resolve, in a better way, some of the sterols, like cholesterol and lathosterol, which are not sufficiently resolved by previously published methods. Its advantages are no need for sample derivatization, good linearity, and the possibility to obtain sterol quantification with isolation in less than 24 h, within a single LC-MS run. For more detailed sterol and oxysterol analyses, methods like those described in McDonald et al. can be used [23,26] alone or in combination with our chromatographic method. Being isocratic, the method can also be easily adapted for preparative chromatography.

## Figures and Tables

**Table 1:** Validation data for sterol intermediates for all measured sterols.

Sterols	Accuracy (%)	Repeatability (% RSD)	Regression Coefficient	Stability 48 h (%)	LOQ (ng/mL)	LOD (ng/mL)	S/N (125 ng/mL)
Zymosterol	105.0	7.5	0.9999	86.6	90.6	27.2	13.8
Dihydrolanosterol	97.9	4.2	0.9999	96.2	148.8	44.6	8.4
Zymostenol	106.4	3.5	0.9999	95.2	78.1	23.4	16.0
FFMAS	86.5	6.1	0.9999	82.5	178.5	53.6	7.0
TMAS	87.2	4.3	0.9998	82.2	187.7	56.3	6.7
Lanosterol	95.7	5.8	0.9998	92.5	115.7	34.7	10.8
7-dehydrodesmosterol	109.7	7.4	0.9996	88.3	152.4	45.7	8.2
24-dehydrolathosterol	103.1	9.4	0.9997	93.0	96.9	29.1	12.9
Desmosterol	96.1	4.5	0.9997	85.4	151.5	45.5	8.3
Lathosterol	101.2	5.2	0.9997	89.4	183.8	55.1	6.8

LOD, limit of detection; LOQ, limit of quantitation; S/N, signal to noise ratio. Linearity was tested in the concentration range from 67.5 to 5000 ng/mL Data validation method is explained in Section 3.4. Quantitation, Accuracy, Repeatability, Linearity, and Stability.

**Table 2:** Concentration of sterols isolated from HepG2 and HepG2 DHCR24 KO cell cultures.

Sterol Measured	HepG2 *n* = 3		HepG2 DHCR24 KO *n* = 3		Statistical Significance with Unpaired *t*-Test *
	Concentration				
	Mean (ng/mL)	SD	Mean (ng/mL)	SD	
**Zymosterol**	192.8	11.7	2446.1	707.1	***
**Dihydrolanosterol**	38.1	3.0	0.0	0.0	****
**Zymostenol**	365.5	38.1	25.8	23.5	**
**T-MAS**	148.5	7.6	1535.9	335.6	**
**Lanosterol**	73.1	3.8	305.9	65.1	**
**7-dehydrodesmosterol**	36.0	2.3	1411.6	236.3	***
**24-dehydrolathosterol**	57.0	3.2	1414.0	236.8	***
**Desmosterol**	248.7	19.8	108156.4	19170.1	***
**Lathosterol**	842.9	152.4	0.0	0.0	***

Mean and SD were calculated from concentrations measured in three biological replicates (details in 4.2 Sample preparation). Statistical significance comparing HepG2 and HepG2 DHCR24 KO with unpaired *t*-test * *p* < 0.05, ** *p* < 0.01, *** *p* < 0.001, **** *p* < 0.0001.

**Table 3:** MS detection conditions and retention times.

Trivial Name	Avanti Polar Lipids Number *	Chemical Name	Molar Mass	MRM *	t_R_ (min)
**Lanosterol**	700063P	8,24-lanostadien-3β-ol	426.72	409/191	21.4
**Dihydro** **lanosterol**	700067P	24,25-dihydrolanosterol	428.73	411/191	26.3
**FFMAS**	700077P	14-demethyl-14-dehydrolanosterol	410.68	393/214	19.5
**TMAS**	700073P	4,4-dimethylcholest-8(9),24-dien-3β-ol	412.69	395/243	20.7
**Zymosterol**	700068P	5α-cholesta-8,24-dien- 3β-ol	384.64	367/215	15.3
**Zymostenol**	700118P	5α-cholest-8-en-3β-ol	386.65	369/215	18.2
**24-dehydro** **lathosterol**	700114P	5α-cholesta-7,24-dien- 3β-ol	384.64	367/215	16.0
**lathosterol**	700069P	5α-cholest-7-en-3β-ol	386.65	369/215	19.1
**7-dehydro** **desmosterol**	700138P	7-dehydrodesmosterol	382.62	365/199	16.3
**Desmosterol**	700060P	3β-hydroxy-5,24-cholestadiene	384.64	367/215	16.9
**Lathosterol-d7**	700056P	5α-Cholest-7-en-3β-ol(25,26,26,26,27,27,27-d7)	393.70	376/215	19.2
**Cholesterol**	Merck (C3045)	Cholest-5-en-3β-ol	386.70	369/215	19.8

* All additional data about standards, including molecular formula, are available on the Avanti Polar Lipids web page (https://avantilipids.com/).
